# A delayed plant disease model with Caputo fractional derivatives

**DOI:** 10.1186/s13662-022-03684-x

**Published:** 2022-01-29

**Authors:** Pushpendra Kumar, Dumitru Baleanu, Vedat Suat Erturk, Mustafa Inc, V. Govindaraj

**Affiliations:** 1 Department of Mathematics, National Institute of Technology Puducherry, Karaikal, 609609 India; 2 Department of Mathematics, Cankaya University, Ankara, Turkey; 3 Institute of Space Sciences, Magurele-Bucharest, R76900 Romania; 4 Department of Mathematics, Ondokuz Mayis University, Atakum, 55200 Samsun Turkey; 5 Department of Computer Engineering, Biruni Universiity, Istanbul, Turkey; 6 Department of Mathematics, Science Faculty, Firat University, Elazig, 23119 Turkey; 7 Department of Medical Research, China Medical University Hospital, China Medical University, Taichung, Taiwan

**Keywords:** 26A33, 34C60, 65D05, 65D30, 65L07, 92C80, 92D40, Fractional mathematical model, Crowding effect, Disease resistance, Incubation period, Caputo fractional derivative, Predictor–corrector algorithm, Time-delay

## Abstract

We analyze a time-delay Caputo-type fractional mathematical model containing the infection rate of Beddington–DeAngelis functional response to study the structure of a vector-borne plant epidemic. We prove the unique global solution existence for the given delay mathematical model by using fixed point results. We use the Adams–Bashforth–Moulton P-C algorithm for solving the given dynamical model. We give a number of graphical interpretations of the proposed solution. A number of novel results are demonstrated from the given practical and theoretical observations. By using 3-D plots we observe the variations in the flatness of our plots when the fractional order varies. The role of time delay on the proposed plant disease dynamics and the effects of infection rate in the population of susceptible and infectious classes are investigated. The main motivation of this research study is examining the dynamics of the vector-borne epidemic in the sense of fractional derivatives under memory effects. This study is an example of how the fractional derivatives are useful in plant epidemiology. The application of Caputo derivative with equal dimensionality includes the memory in the model, which is the main novelty of this study.

## Introduction

Plant epidemiology is the branch of science in which we study various diseases in different families of plants. A plant has to keep faith simply on cellular inborn immunity to bargain with infections as it does not hold any shape of mobile protection, and therefore it demonstrates many plant-exclusive behaviors [1]. A one of the plant viral epidemic, called vector-borne diseases, has exerted the attention of scientists doing research via mathematical modeling [2, 3]. Mathematical models are becoming very effective to utilize the dynamics of vector-borne plant epidemic transmission in host plants. Some effective optimal controls can be summarized via these frameworks [4]. Various mathematical models have been utilized by the mathematicians to give a framework of particular disease modelings. In this series the dynamics of Jatropha curcas mosaic epidemic, which is spread by whitefly vectors, is studied mathematically in [5] by observing oscillations in the model due to a large rate of infection. Also, a structure of soil borne plant epidemic along with host demography by showing limit cycle nature has framed in [6]. In the modeling studies, researchers have been taken bilinear infection transmission. On the other side, in [7, 8] a nonlinear rate of incidence is taken for defining the vector-borne plant epidemic propagation. Here we have another form of modeling defined by including time delay in the biological systems. The delay vector-borne plant epidemic systems can specify periodic oscillations, stability switches, transcritical bifurcation, etc. [5, 6]. Such observations are little crucial to estimate the firmness of infection and control of disease. Zhang et al. [9] revised a plant disease model given in [10] by taking the plant incubation duration as a delay to justify the necessary changes in the given dynamical structure. Also, a mathematical framework for the structure of soil-borne plant epidemic by taking the delay in time cause of the latent period of vectors/inoculum is given in [6]. The modification in a model given in [11] made by Jackson [7] for specifying the vector-borne epidemic dynamics in plants by analyzing multiple delays to include the latent period in vectors and incubation periods of plants.

Nowadays, fractional calculus is a very well-known phenomenon in the field of mathematical modeling. In this tool, there are many fractional-order derivatives presented for applying in modeling. A large study on theory and applications of fractional-order derivatives have been done by researchers [12–14]. Some specific studies on fractional-order Lotka–Volterra population model [15], population structure of two interacting species [16], nonclassical type model for the spreading of pests in tea plants [17], fractional optimal control techniques [18], nonclassical chemical kinetics system [19], dynamics of SEIR model of measles [20], immunogenetic tumor model [21], new technique to solve noninteger-order PDEs [22] have been proposed. Sene et al. [23] has analyzed a four-dimensional hyperchaotic system in the sense of Caputo-type fractional derivative. A clear role of vaccine in the Covid-19 epidemic can be learned by using a fractional-order SEIR model from [24]. In [25] the authors explored the dynamics of the mosaic disease via a nonclassical mathematical model. Moreover, some studies on CDV and rabies epidemics [26], oncolytic virotherapy [27], and huanglongbing transmission [28] have been recently explored. Nonclassical derivatives have been regularly used to study the structures of various deathly diseases. Recently, a number of researchers have used different fractional derivatives in epidemiology for analyzing the structure of coronavirus [29–31], malaria [32], and tuberculosis [33]. A Mittag-Leffler kernel-type SIR disease model is given in [34]. In [35] the authors have analyzed a fractional-order predator–prey model. In [36] a stochastic approach to derive the dynamics of a Covid-19 disease model has been used. Atangana [37] has modeled the transmission of Covid-19 by using fractal-fractional operators. One of the early applications of new generalized Caputo-type noninteger-order derivative in ecology is given in [38]. An example of importance of fractional derivatives in physics is presented in [39]. Fractional differential equations have both delay and nondelay cases, and the derivatives of such nonclassical type are smoothly used to study them. In this paper, we apply the well-known Caputo fractional derivatives with singular type memory for studying the proposed time-delay plant epidemic model.

The current paper is organized as follows. In the preliminaries Sect. 2, we recall the definition of Caputo fractional derivative along with specifying the single-parameter form of the Mittag-Leffler function. In Sect. 3, we specify the structure of integer-order plant disease model proposed by Basir et al. [40]. Here we remind the corresponding basic reproductive number and the theorems on stability of disease-free equilibriums of the dynamical model. We reformulate the classical model in a fractional-order Caputo model by giving the motivation of such changes because we believe that the fractional derivatives better fit real-word phenomena. Section 4 is entitled with mathematical analysis of the fractional model. In this section, we made some parts for existence of the unique global solution for the time-delay Caputo modeling by using fixed-point theory. In the other part of this section, we derive the solution of the model by using an efficient delay-type numerical algorithm. As we know, when we study any real-world phenomena or, more specifically, the dynamics of any epidemic, some common concerns always exist, for example, how the disease will behave for the long time interval or how we can project the real data for future predictions? To fulfil these requirements, the graphical interpretations are very important. In Sect. 5, we establish a sufficient discussion on the graphical analysis for the proposed model by using specific numerical values of the significant parameters. We evaluate the role of infection rate and the time delay by using their various values for the given time period. In the graphical structures, we give some 2D compatible plots and some 3D graphics by the help of Python software. Finally, we conclude our all results by giving a smooth finish to our study.

## Preliminaries

### Definition 1

([14])

The Caputo fractional derivative of a function $\mathcal{Y}\in C^{\alpha }_{-1}$ is given by

1 $$ D^{\lambda }_{t}\mathcal{Y} (t )= \textstyle\begin{cases} \frac{d^{\alpha }\mathcal{Y} (t )}{dt^{\alpha }},& \lambda =\alpha \in \mathbb{N}, \\ \frac{1}{\Gamma (\alpha -\lambda )}\int ^{t}_{a}{{ (t-\vartheta )}^{\alpha -\lambda -1}\mathcal{Y}^{(\alpha )} ( \vartheta )\,d\vartheta },& \alpha -1< \lambda < \alpha , \alpha \in \mathbb{N}. \end{cases} $$

### Definition 2

([14])

The Riemann–Liouville fractional integral of a function $\mathcal{Y}\in C^{\alpha }_{-1}$ is given by

2 $$ J^{\lambda }\mathcal{Y}(t)=\frac{1}{\Gamma (\lambda )} \int ^{t}_{a}{{(t- s )}^{\lambda -1} \mathcal{Y}(s )\,ds }. $$

### Definition 3

([14])

The one-parameter form of the Mittag-Leffler function is defined as

3 $$ E_{\lambda } (z )=\sum^{\infty }_{\varpi =0}{ \frac{z^{\varpi }}{\Gamma (\lambda \varpi +1 )}},\quad \lambda >0, z\in \mathbb{C}. $$

## Model structure

In plant epidemiology, a number of deathly diseases or viruses have been observed, which are becoming very harmful for our plants. In the mathematical point of view, some models have been analyzed to study the dynamics of these diseases like mosaic disease [41], huanglongbing virus transmission within a citrus tree [42], Xylella fastidiosa epidemic in olive trees [43], etc. In this paper, we adopt a mathematical delay model proposed by Basir et al. [40] for defining the structure of vector-borne plant disease. In the model the authors specified three different classes; plenty of susceptible plant $x(t)$, infected plants $y(t)$, and virus carrier or infected vector $v(t)$. The authors used the dimensions m^−2^ for $x(t)$, $y(t)$ and plant^−1^ for $v(t)$. The integer-order model is as follows:

4 $$ \textstyle\begin{cases} x'(t)= g x [1- \frac{x+ y}{k} ] - \frac{\Omega x v}{1+ \delta x+ b v}, \\ y'(t)= \frac{\Omega e^{-\beta \tau } x(t- \tau ) v(t- \tau )}{1+ \delta x(t- \tau )+ b v(t- \tau )}- (\beta + \Lambda ) y, \\ v'(t)= \alpha y- \gamma v. \end{cases} $$

In this model, the authors used the Beddington–DeAngelis-type infection rate $\frac{\Omega x v}{1+ \delta x+ b v}$. Here the plant resistance rate is denoted by *δ*, and the crowding effect rate of vectors is given by *b*. The term $\frac{\Omega e^{-\beta \tau } x(t- \tau ) v(t- \tau )}{1+ \delta x(t- \tau )+ b v(t- \tau )}$ is the effect of time delay in the form of incubation period of the plant. The basic reproduction number is calculated by $\mathcal{R}_{0}= \frac{k\Omega e^{-\beta \tau }\alpha }{\gamma (\beta + \Lambda )(1+ \delta k)}$ (independent from the crowding effect *b*). The disease-free equilibrium (DFE) is calculated by $E_{1}(k, 0, 0)$.

Further properties of the given model related to the disease-free and endemic equilibrium point stability, nonnegativity, boundedness of solutions, and model origin can be studied from [40]. Table 1 is devoted to the parameter description and their numerical values used in the practical simulations. Since early decade, a number of nonclassical type derivatives have been proposed and applied by many mathematicians, where the Caputo fractional derivative is derived in the sense of singular-type kernel. In this part, we reformulate the given integer-order model (4) into the Caputo sense. The main reason or motivation of this replacement is to explore the dynamics of given integer-order model at fractional-order values to simulate the memory effects. So the generalization of the given model (4) into the Caputo-type model along with taking the equal dimension time$^{-\lambda }$ on both sides is given as follows:

5 $$ \textstyle\begin{cases} ^{C}D_{t}^{\lambda }x(t)= g^{\lambda }x [1- \frac{x+ y}{k^{\lambda }} ] - \frac{\Omega ^{\lambda }x v}{1+ \delta ^{\lambda }x+ b^{\lambda }v}, \\ ^{C}D_{t}^{\lambda }y(t)= \frac{\Omega ^{\lambda }e^{-\beta \tau } x(t- \tau ) v(t- \tau )}{1+ \delta ^{\lambda }x(t- \tau )+ b^{\lambda }v(t- \tau )}- (\beta ^{\lambda }+ \Lambda ^{\lambda }) y, \\ ^{C}D_{t}^{\lambda }v(t)= \alpha ^{\lambda }y- \gamma ^{\lambda }v, \end{cases} $$

where $^{C}D_{t}^{\lambda }$ is the Caputo derivative operator of fractional order *λ*. The above model can be written in its equivalent form by specifying three different singular type kernels $\mathcal{Z}_{1}$, $\mathcal{Z}_{2}$, $\mathcal{Z}_{3}$ as follows:

6 $$ \textstyle\begin{cases} ^{C}D_{t}^{\lambda }x(t)= \mathcal{Z}_{1}(t, x, x-\tau ), \\ ^{C}D_{t}^{\lambda }y(t)= \mathcal{Z}_{2}(t, y, y-\tau ), \\ ^{C}D_{t}^{\lambda }v(t)= \mathcal{Z}_{3}(t, z, z-\tau ), \end{cases} $$

where $\mathcal{Z}_{1}$, $\mathcal{Z}_{2}$, $\mathcal{Z}_{3}$ are the respective singular kernels for the respective model equations $x(t)$, $y(t)$. $v(t)$ (equal to the right-hand sides of model (5)).

**Table 1:** Identification of model parameters [40]

Parameter	Identification	Values
*g*	growth rate of plant density	0.1 day^−1^
Ω	infection rate of plant	0.4 vector^−1^ day^−1^
*k*	maximum plant density	1 m^−2^
*α*	growth rate of infected vector	0.4 day^−1^
*β*	infected plant removal rate	0.1 day^−1^
*δ*	resistance rate of plant	0.5 m^2^
Λ	additional death due to infection	0.025 day^−1^
*γ*	mortality rate of vector	0.1 day^−1^
*b*	crowding effect of vector	0.5 plant
*τ*	delay in time	[0,6]

### Some stability results

To state some analysis related to the stability of the disease-free equilibrium points for given fractional-order system, we analyze the linearization of system (5) at equilibrium point $E(x_{*}, y_{*}, v_{*})$ as follows:

7 $$ \textstyle\begin{cases} ^{C}D_{t}^{\lambda }x(t)= g^{\lambda }x [1- \frac{2x_{*}+ y_{*}}{k^{\lambda }} ] - \frac{\Omega ^{\lambda }x v_{*}(1+ b^{\lambda }v_{*})}{(1+ \delta ^{\lambda }x_{*}+ b^{\lambda }v_{*})^{2}}- \frac{r^{\lambda }x_{*} y}{k^{\lambda }} - \frac{\Omega ^{\lambda }x_{*} v(1+ \delta ^{\lambda }x_{*})}{(1+ \delta ^{\lambda }x_{*}+ b^{\lambda }v_{*})^{2}}, \\ ^{C}D_{t}^{\lambda }y(t)= \frac{\Omega ^{\lambda }e^{-\beta \tau }(1+ b^{\lambda }v_{*}) x(t- \tau ) v_{*}}{(1+ \delta ^{\lambda }x_{*}+ b^{\lambda }v_{*})^{2}}+ \frac{\Omega ^{\lambda }e^{-\beta \tau }(1+ \delta ^{\lambda }x_{*}) v(t- \tau ) x_{*}}{(1+ \delta ^{\lambda }x_{*}+ b^{\lambda }v_{*})^{2}} - (\beta ^{\lambda }+ \Lambda ^{\lambda }) y, \\ ^{C}D_{t}^{\lambda }v(t)= \alpha ^{\lambda }y- \gamma ^{\lambda }v. \end{cases} $$

Taking the Laplace transform of both sides of system (7) gives

8 $$ \textstyle\begin{cases} s^{\lambda }\mathcal{L}[x(s)]- s^{\lambda -1}x(0)= g^{\lambda } [1- \frac{2x_{*}+ y_{*}}{k^{\lambda }} ]\mathcal{L}[x(s)] - \frac{\Omega ^{\lambda }v_{*}(1+ b^{\lambda }v_{*})}{(1+ \delta ^{\lambda }x_{*}+ b^{\lambda }v_{*})^{2}} \mathcal{L}[x(s)]\\ \hphantom{s^{\lambda }\mathcal{L}[x(s)]- s^{\lambda -1}x(0)=}{} - \frac{r^{\lambda }x_{*}}{k^{\lambda }}\mathcal{L}[y(s)] - \frac{\Omega ^{\lambda }x_{*} (1+ \delta ^{\lambda }x_{*})}{(1+ \delta ^{\lambda }x_{*}+ b^{\lambda }v_{*})^{2}} \mathcal{L}[v(s)], \\ s^{\lambda }\mathcal{L}[y(s)]- s^{\lambda -1}y(0)= \frac{\Omega ^{\lambda }e^{-\beta \tau }(1+ b^{\lambda }v_{*}) v_{*}}{(1+ \delta ^{\lambda }x_{*}+ b^{\lambda }v_{*})^{2}}e^{-s \tau } (\mathcal{L}[x(s)]+ \int _{-\tau }^{0}e^{-st}\phi (t)\,dt ) \\ \hphantom{s^{\lambda }\mathcal{L}[y(s)]- s^{\lambda -1}y(0)=}{}+ \frac{\Omega ^{\lambda }e^{-\beta \tau }(1+ \delta ^{\lambda }x_{*}) x_{*}}{(1+ \delta ^{\lambda }x_{*}+ b^{\lambda }v_{*})^{2}}e^{-s \tau } (\mathcal{L}[v(s)]+ \int _{-\tau }^{0}e^{-st}\phi (t)\,dt )\\ \hphantom{s^{\lambda }\mathcal{L}[y(s)]- s^{\lambda -1}y(0)=}{} - (\beta ^{\lambda }+ \Lambda ^{\lambda }) \mathcal{L}[y(s)], \\ s^{\lambda }\mathcal{L}[v(s)]- s^{\lambda -1}v(0)= \alpha ^{\lambda }\mathcal{L}[y(s)]- \gamma ^{\lambda }\mathcal{L}[v(s)], \end{cases} $$

where $\mathcal{L}[x(s)]$, $\mathcal{L}[y(s)]$, and $\mathcal{L}[v(s)]$ are the Laplace transforms of $x(t)$, $y(t)$, and $v(t)$. System (7) can be rewritten as

$$ \Delta (s) \cdot \begin{bmatrix} \mathcal{L}[x(s)] \\ \mathcal{L}[y(s)] \\ \mathcal{L}[v(s)] \end{bmatrix} = \begin{bmatrix} \eta _{1}(s) \\ \eta _{2}(s) \\ \eta _{3}(s) \end{bmatrix}, $$

where

9 $$\begin{aligned}& \textstyle\begin{cases} \eta _{1}(s)= s^{\lambda -1}x(0), \\ \eta _{2}(s)= s^{\lambda -1}y(0)+ \frac{\Omega ^{\lambda }e^{-\beta \tau }(1+ b^{\lambda }v_{*}) v_{*}}{(1+ \delta ^{\lambda }x_{*}+ b^{\lambda }v_{*})^{2}}e^{-s \tau } \int _{-\tau }^{0}e^{-st}\phi (t)\,dt \\ \hphantom{\eta _{2}(s)=}{}+ \frac{\Omega ^{\lambda }e^{-\beta \tau }(1+ \delta ^{\lambda }x_{*}) x_{*}}{(1+ \delta ^{\lambda }x_{*}+ b^{\lambda }v_{*})^{2}}e^{-s \tau } \int _{-\tau }^{0}e^{-st}\phi (t)\,dt, \\ \eta _{3}(s)= s^{\lambda -1}v(0), \end{cases}\displaystyle \\& \Delta (s)= \begin{bmatrix} s^{\lambda }- g^{\lambda } (1-\frac{2x_{*}+y_{*}}{k^{\lambda }} )+ \frac{\Omega ^{\lambda }v_{*}(1+ b^{\lambda }v_{*})}{{(1+\delta ^{\lambda }x_{*}+ b^{\lambda }v_{*})}^{2}} & \frac{r^{\lambda }x_{*}}{k^{\lambda }} & \frac{\Omega ^{\lambda }x_{*}(1+ \delta ^{\lambda }x_{*})}{{(1+\delta ^{\lambda }x_{*}+ b^{\lambda }v_{*})}^{2}} \\ - \frac{\Omega ^{\lambda }e^{-\beta \tau } v_{*}(1+ b^{\lambda }v_{*})e^{-s \tau }}{{(1+\delta ^{\lambda }x_{*}+ b^{\lambda }v_{*})}^{2}} & s^{\lambda }+ (\Lambda ^{\lambda }+\beta ^{\lambda }) & - \frac{\Omega ^{\lambda }e^{-\beta \tau } x_{*}(1+ \delta ^{\lambda }x_{*})e^{-s \tau }}{{(1+\delta ^{\lambda }x_{*}+ b^{\lambda }v_{*})}^{2}} \\ 0 & -\alpha ^{\lambda }& s^{\lambda }+\gamma ^{\lambda } \end{bmatrix} , \end{aligned}$$

which is a characteristic matrix of system (7). Now the characteristic matrix of the system at the disease-free equilibrium $E_{1}(k^{\lambda }, 0, 0)$ is given by

$$ \Delta (s)= \begin{bmatrix} s^{\lambda }+ g^{\lambda }& r^{\lambda }& \frac{\Omega ^{\lambda }k^{\lambda }}{(1+\delta ^{\lambda }k^{\lambda })} \\ 0 & s^{\lambda }+ (\Lambda ^{\lambda }+\beta ^{\lambda }) & - \frac{\Omega ^{\lambda }e^{-\beta \tau } k^{\lambda }e^{-s \tau }}{(1+\delta ^{\lambda }k^{\lambda })} \\ 0 & -\alpha ^{\lambda }& s^{\lambda }+\gamma ^{\lambda} \end{bmatrix}. $$

The characteristic equation at $E_{1}(k^{\lambda }, 0, 0)$ is

$$ \det \bigl(\Delta (s)\bigr)= \bigl(s^{\lambda }+ g^{\lambda }\bigr) \biggl[\bigl(s^{\lambda }+ \bigl(\Lambda ^{\lambda }+\beta ^{\lambda }\bigr)\bigr) \bigl(s^{\lambda }+\gamma ^{\lambda } \bigr) - \frac{\alpha ^{\lambda }\Omega ^{\lambda }e^{-\beta \tau } k^{\lambda }e^{-s \tau }}{(1+\delta ^{\lambda }k^{\lambda })} \biggr]=0.$$

Now there are two cases: (i) $\tau = 0$ and (ii) $\tau > 0$. We have the following theorems.

#### Theorem 1

*For the time delay* $\tau = 0$, *the disease*-*free equilibrium* $E_{1}$ *of system* (4) *is stable if* $\mathcal{R}_{0}< 1$ *and unstable if* $\mathcal{R}_{0}> 1$.

#### Proof

For $\tau = 0$, let $B= s^{\lambda }$. Then the characteristic equation is

10 $$ \bigl(B+ g^{\lambda }\bigr)\cdot \biggl(B^{2}+ \bigl(\beta ^{\lambda }+ \Lambda ^{\lambda }+ \gamma ^{\lambda }\bigr)B+ \bigl(\beta ^{\lambda }+ \Lambda ^{\lambda }\bigr)\gamma ^{\lambda }- \frac{\alpha ^{\lambda }\Omega ^{\lambda }k^{\lambda }}{1+ \delta ^{\lambda }k^{\lambda }} \biggr)= 0. $$

Thus at the DFE $E_{1}(k^{\lambda }, 0, 0)$, one eigenvalue is $-g^{\lambda }< 0$, and the other eigenvalues are negative or with negative real part if $(\beta ^{\lambda }+ \Lambda ^{\lambda })\gamma ^{\lambda }- \frac{\alpha ^{\lambda }\Omega ^{\lambda }k^{\lambda }}{1+ \delta ^{\lambda }k^{\lambda }} > 0$, which is equivalent to $\mathcal{R}_{0}< 1$. □

When $\tau >0$, one eigenvalue is $-g^{\lambda }< 0$, and the other roots satisfy the quadratic equation

11 $$ \begin{aligned}[b] Z(s, \tau )&= \bigl(s^{\lambda }+ \bigl(\Lambda ^{\lambda }+\beta ^{\lambda }\bigr)\bigr) \bigl(s^{\lambda }+\gamma ^{\lambda }\bigr) - \frac{\alpha ^{\lambda }\Omega ^{\lambda }e^{-\beta \tau } k^{\lambda }e^{-s \tau }}{(1+\delta ^{\lambda }k^{\lambda })} \\ &= s^{2\lambda }+ \bigl(\beta ^{\lambda }+ \Lambda ^{\lambda }+ \gamma ^{\lambda }\bigr)s^{\lambda }+ \bigl(\beta ^{\lambda }+ \Lambda ^{\lambda }\bigr)\gamma ^{\lambda }- \frac{\alpha ^{\lambda }\Omega ^{\lambda }k^{\lambda }e^{-(\beta +s)\tau }}{1+ \delta ^{\lambda }k^{\lambda }}= 0. \end{aligned} $$

We study the following case.

#### Theorem 2

*DFE* $E_{1}$ *of system* (5) *for* $\tau > 0$ *is asymptotically stable for* $\mathcal{R}_{0} < 1$ *and unstable for* $\mathcal{R}_{0} > 1$.

#### Proof

Assume that $\mathcal{R}_{0} > 1$. Then $Z(0, \tau )= (\beta ^{\lambda }+ \Lambda ^{\lambda })\gamma ^{\lambda }- \frac{\alpha ^{\lambda }\Omega ^{\lambda }k^{\lambda }e^{-\beta \tau }}{1+ \delta ^{\lambda }k^{\lambda }}= (\beta ^{\lambda }+ \Lambda ^{\lambda })\gamma ^{\lambda }(1- \mathcal{R}_{0})< 0$. Since $\lim_{s\rightarrow \infty } Z(s, \tau )= \infty $, there exists at least one real-positive root of the characteristic equation (11), which gives that $E_{1}$ is unstable.

Now let $\mathcal{R}_{0} < 1$. We have to prove that for $\tau > 0$, none of the characteristic roots can approach the imaginary axis. Assume by contradiction that for some $\tau > 0$, $s^{\lambda }= i\theta ^{\lambda }$ is a root of (11). Taking $s^{\lambda }= i\theta ^{\lambda }$ in (11) and splitting real and imaginary parts, we get

12 $$ \begin{aligned} & -\theta ^{2\lambda } + M_{2} = M_{3} \cos \theta ^{\lambda }\tau , \\ & \theta ^{\lambda }M_{1}= -M_{3}\sin \theta ^{\lambda }\tau , \end{aligned} $$

where $M_{1}= (\beta ^{\lambda }+ \Lambda ^{\lambda }+ \gamma ^{\lambda })$, $M_{2}= (\beta ^{\lambda }+ \Lambda ^{\lambda })\gamma ^{\lambda }$, and $M_{3}= \frac{\alpha ^{\lambda }\Omega ^{\lambda }k^{\lambda }e^{-\beta \tau }}{1+ \delta ^{\lambda }k^{\lambda }}$. Taking the square and adding to the above equations, we finally get

13 $$ \theta ^{4\lambda } + \theta ^{2\lambda }\bigl(\bigl(\beta ^{\lambda }+ \Lambda ^{ \lambda }\bigr)^{2}+ \gamma ^{2\lambda }\bigr)+ \bigl(M_{2}^{2} - M_{3}^{2}\bigr)=0. $$

Note that $\mathcal{R}_{0}< 1$ implies $M_{3} < M_{2}$. Since the above equation has no real roots for *θ*, the characteristic equation (11) cannot have purely imaginary roots. Thus, for $\mathcal{R}_{0}< 1$, the steady state $E_{1}$ is asymptotically stable for all $\tau \geq 0$. □

## Mathematical analysis of the Caputo-type model

### Existence and uniqueness of the solution

Many research works are available in the literature where the existence and uniqueness of solution for nondelay-type fractional differential equations are proved. In comparison, there are less proofs of the existence of a unique solution for the delay-type fractional-order initial value problems. A number of researchers have proposed their ideas on this topic. In this part of the paper, we prove the existence and uniqueness of a solution for the proposed singular fractional time-delay plant disease model by using the ideas of Cong and Tuan et al. [44], who have proved the results by applying fixed point theory. Also, the same results have been used by Kumar and Erturk [30] to simulate a coronavirus time-delay dynamical model. Now we derive the proofs for the equation system $\mathcal{Y}(t)= (x(t), y(t), v(t)$ with kernels $\mathcal{B}(\mathcal{Y}(t), \mathcal{Y}(t- \tau ))= \mathcal{Z}_{1}(t, x, x-\tau ), \mathcal{Z}_{2}(t, y, y-\tau ), \mathcal{Z}_{3}(t, v, v- \tau )$. Let us consider the singular type noninteger-order delay initial value problem (IVP)

14 $$ ^{C}D_{t}^{\lambda } \mathcal{Y}(t)= \mathcal{B}\bigl(t, \mathcal{Y}(t), \mathcal{Y}(t- \tau )\bigr), \quad t\in [0, T], 0< \lambda \leq 1, $$

with initial condition

15 $$ \mathcal{Y}(t)= k_{1},\quad t\in [-\tau , 0], $$

where $\mathcal{Y}\in \mathbb{R}^{n}$, $T > 0$, and $\mathcal{B}: [0, T] \times \mathbb{R}^{n}\times \mathbb{R}^{n} \rightarrow \mathbb{R}^{n}$ is continuous.

($\mathbb{R}^{n}$ is the *n*-dimensional Euclidean space with norm $\|\cdot \|$)

#### Lemma 1

([44])

*The mapping* $\mho \in C([-\tau , T]; \mathbb{R}^{n})$, *where* $C([-\tau , T]; \mathbb{R}^{n})$ *is a space of continuous mappings* ℧ *from* $[-\tau , T]$ *to* $\mathbb{R}^{n}$ *with the supremum norm* ${\| \cdot \| }_{\infty })$, *is a solution to the IVP* (14)*–*(15) *on the interval* $[-\tau , T]$ *if and only if it solves the fractional*-*order time*-*delay integral equation*

16 $$ \mathcal{Y}(t)= \mathcal{Y}(0)+ \frac{1}{\Gamma (\lambda )} \int _{0}^{t}{(t- \zeta )}^{\lambda -1} \mathcal{B}\bigl(\zeta , \mathcal{Y}(\zeta ), \mathcal{Y}(\zeta - \tau )\bigr)\,d\zeta \quad \forall t\in [0, T], $$

*with initial values*

17 $$ \mathcal{Y}(t)= k_{1}, \quad t\in [-\tau , 0]. $$

*Note:* [44] In the given exclusive proof modus, we did not consider whether the time-delay variable of $\mathcal{B}$ satisfies the Lipschitz condition or not. We only considered the satisfaction of the Lipschitz property of $\mathcal{B}$ for the delay-free parameter *t*.

#### Theorem 3

(Existence of a unique global solution)

*Suppose that* $\mathcal{B}: [0, T]\times \mathbb{R}^{n} \times \mathbb{R}^{n} \rightarrow \mathbb{R}^{n}$ *is a continuous mapping satisfying the Lipschitz property with respect to the nondelay variable and that there exists a continuous nonnegative function* $L : [0, T] \times \mathbb{R}^{n} \rightarrow \mathbb{R}_{\geq 0}$ *such that*

18 $$ \bigl\Vert \mathcal{B}(t, \mathcal{Y}, \mathcal{Y}_{d})- \mathcal{B}(t, \mathcal{Y}_{1}, \mathcal{Y}_{d}) \bigr\Vert \leq L(t, \mathcal{Y}_{d}) \Vert \mathcal{Y}- \mathcal{Y}_{1} \Vert $$

*for all* $t \in [0, T]$ *and* $\mathcal{Y}, \mathcal{Y}_{d}, \mathcal{Y}_{1} \in \mathbb{R}^{n}$. *Then the IVP* (14)*–*(15) *has a unique global solution* ℧ *on the time interval* $[-\tau , T]$.

#### Proof

By Lemma (1) Eqs. (14)–(15) are equivalent to the IVP (16)–(17). Firstly, we consider the case $0 < T \leq \tau $. In that case, Eq. (16) has the form

$$ \mathcal{Y}(t)= \mathcal{Y}(0)+ \frac{1}{\Gamma (\lambda )} \int _{0}^{t}{(t- \zeta )}^{\lambda -1} \mathcal{B}\bigl(\zeta , \mathcal{Y}(\zeta ), k_{1}\bigr)\,d \zeta \quad \forall t\in [0, T] $$

by Tisdell [45, Theorem 6.4, p. 310]. This integral equation has a unique solution on the interval $[0, T]$. We denote this solution by $\varpi _{\tau }^{*}$ and take

19 $$\begin{aligned} \mho _{T}(t,k_{1}) := \textstyle\begin{cases} k_{1}, & t\in [-\tau ,0], \\ \varpi _{\tau }^{*}(t), & t\in [0, T]. \end{cases}\displaystyle \end{aligned}$$

Then $\mho _{T}(t, k_{1})$ is the unique solution of Eqs. (16)–(17) on $[-\tau , T]$.

In the other case where $T > \tau $, we break the interval $[0, T]$ into $[0, \tau ]\cup \cdots \cup [(a_{0} -1)\tau , a_{0}\tau ] \cup [a_{0} \tau , T]$, where $a_{0}\in \mathbb{N}$ and $0\leq T- a_{0}\tau < \tau $. For the interval $[-\tau , \tau ]$, in a similar way as above, we write a unique solution of Eqs. (16)–(17) specified by $\mho _{\tau }$. Now, by using the induction property, we will proof the existence of unique solution on the interval $[-\tau , a_{0}\tau ]$. Now suppose that Eqs. (16)–(17) have a unique solution on the interval $[-\tau , a\tau ]$ for some $1\leq a< a_{0}$. We denote that solution by $\mho _{a\tau }(., k_{1})$. On $[a\tau , (a + 1)\tau ]$, we define the operator $\textit{A}_{(a+1)\tau ,k_{1}}:C([a\tau , (a+1)\tau ]; \mathbb{R}^{n}) \rightarrow C([a\tau , (a+1)\tau ]; \mathbb{R}^{n})$ as follows:

$$\begin{aligned}& (\textit{A}_{(a+1)\tau ,k_{1}}\varpi ) (t)\\& \quad := \mathcal{Y}(0)+ \frac{1}{\Gamma (\lambda )} \int _{0}^{a\tau }{(t- \zeta )}^{\lambda -1} \mathcal{B}\bigl(\zeta , \mho _{a\tau }(\zeta , k_{1}), \mho _{a\tau }( \zeta - \tau , k_{1})\bigr)\,d\zeta \\& \qquad {}+ \frac{1}{\Gamma (\lambda )} \int _{a\tau }^{t}{(t- \zeta )}^{ \lambda -1} \mathcal{B}\bigl(\zeta , \varpi (\zeta ), \mho _{a\tau }(\zeta - \tau , k_{1})\bigr)\,d\zeta \quad \forall t\in \bigl[a\tau , (a+1)\tau \bigr]. \end{aligned}$$

Let $\beta _{a}$ be a positive constant satisfying $\beta _{a}> 2\max_{t\in [a\tau ,(a+1)\tau ]}L(t,\mho _{a\tau }(t- \tau , k_{1}))$. On the space $C([a\tau , (a+1)\tau ]; \mathbb{R}^{n})$, we define the new metric

$$ d_{\beta a}(\varpi , \varpi _{1}):= \sup _{t\in [a\tau ,(a+1)\tau ]} \frac{ \Vert \varpi (t)- \varpi _{1}(t) \Vert }{E_{\lambda }(\beta _{a}t^{\lambda })}\quad \forall \varrho ,\varrho _{1}\in C\bigl(\bigl[a\tau , (a+1)\tau \bigr]:\mathbb{R}^{n} \bigr),$$

where $E_{\lambda }:\mathbb{R}\rightarrow \mathbb{R}$ is the Mittag-Leffler function (3). Then the space $C([a\tau , (a+1)\tau ];\mathbb{R}^{n})$ equipped with the metric $d_{\beta a}$ is complete. Next, we will prove that the operator $\textit{A}_{(a+1)\tau ,k_{1}}$ is contractive on $(C([a\tau , (a+1)\tau ];\mathbb{R}^{n}), d_{\beta _{a}})$. Indeed, for all $\varpi , \varpi _{1}\in C([a\tau , (a+1)\tau ];\mathbb{R}^{n})$ and $t\in [a\tau , (a+1)\tau ]$, we have

20 $$ \begin{gathered}[b] \bigl\Vert (\textit{A}_{(a+1)\tau ,k_{1}}\varpi ) (t)- ( \textit{A}_{(a+1)\tau ,k_{1}}\varpi _{1}) (t) \bigr\Vert \\ \quad \leq \frac{\max_{t\in [a\tau ,(a+1)\tau ]}L(t, \mho _{a\tau }(t- \tau , k_{1}))}{\Gamma (\lambda )} \int _{a\tau }^{t}(t- \zeta )^{\lambda - 1} \bigl\Vert \varpi (\zeta )- \varpi _{1}(\zeta ) \bigr\Vert \,d\zeta \\ \quad \leq \frac{\max_{t\in [a\tau ,(a+1)\tau ]}L(t, \mho _{a\tau }(t- \tau , k_{1}))}{\Gamma (\lambda )}\\ \qquad {}\times\int _{a\tau }^{t}(t- \zeta )^{\lambda - 1} E_{\lambda }\bigl(\beta _{a} \zeta ^{\lambda }\bigr) \frac{ \Vert \varpi (\zeta )- \varpi _{1}(\zeta ) \Vert }{E_{\lambda }(\beta _{a} \zeta ^{\lambda })} \,d\zeta . \end{gathered} $$

This implies that

21 $$ \begin{aligned}[b] & \frac{ \Vert (\textit{A}_{(a)\tau ,k_{1}}\varpi )(t)- (\textit{A}_{(a)\tau ,k_{1}}\varpi _{1})(t) \Vert }{E_{\lambda }(\beta _{a} t^{\lambda })} \\ &\quad \leq \frac{\max_{t\in [a\tau ,(a+1)\tau ]}L(t, \mho _{a\tau }(t- \tau , k_{1}))}{E_{\lambda }(\beta _{a} t^{\lambda })}d_{ \beta a}(\varpi ,\varpi _{1}) \frac{1}{\Gamma (\lambda )} \int _{a\tau }^{t}(t- \zeta )^{\lambda - 1} E_{\lambda }\bigl(\beta _{a} \zeta ^{\lambda }\bigr) \,d \zeta \\ &\quad \leq \frac{\max_{t\in [a\tau ,(a+1)\tau ]}L(t, \mho _{a\tau }(t- \tau , k_{1}))}{E_{\lambda }(\beta _{a} t^{\lambda })}d_{ \beta a}(\varpi ,\varpi _{1}) \frac{1}{\Gamma (\lambda )} \int _{0}^{t}(t- \zeta )^{\lambda - 1} E_{\lambda }\bigl(\beta _{a} \zeta ^{\lambda }\bigr) \,d \zeta \\ &\quad \leq \frac{\max_{t\in [a\tau ,(a+1)\tau ]}L(t, \mho _{a\tau }(t- \tau , k_{1}))}{E_{\lambda }(\beta _{a} t^{\lambda })}d_{ \beta a}(\varpi ,\varpi _{1})I_{0}^{\lambda } \biggl({^{C}}D_{0}^{\lambda} \biggl(\frac{E_{\lambda }(\beta _{a}t^{\lambda })}{\beta _{a}} \biggr) \biggr) \\ &\quad \leq \frac{\max_{t\in [a\tau ,(a+1)\tau ]}L(t, \mho _{a\tau }(t- \tau , k_{1}))}{\beta _{a}}d_{ \beta a}(\varpi ,\varpi _{1}) \end{aligned} $$

for all $t\in [a\tau , (a+1)\tau ]$. Therefore

22 $$ \begin{aligned}[b] d_{\beta _{a}}(\textit{A}_{(a+1)\tau ,k_{1}}\varpi , \textit{A}_{(a+1)\tau ,k_{1}}\varpi _{1}) &\leq \frac{\max_{t\in [a\tau ,(a+1)\tau ]}L(t, \mho _{a\tau }(t- \tau , k_{1}))}{\beta _{a}}d_{ \beta a}( \varpi ,\varpi _{1}) \\ & \leq \frac{1}{2}d_{\beta _{a}}(\varpi , \varpi _{1}) \end{aligned} $$

for all $\varpi , \varpi _{1}\in C([a\tau , (a+1)\tau ];\mathbb{R}^{n})$. By the Banach fixed-point theorem, of $\textit{A}_{(a+1)\tau ,k_{1}}$ has a unique fixed-point $\varpi ^{*}_{(a+1)\tau }$ in $C([a\tau , (a+1)\tau ];\mathbb{R}^{n})$. Put

23 $$\begin{aligned} \mho _{(a+1)\tau }(t,k_{1}) := \textstyle\begin{cases} \mho _{a\tau (t,k_{1})}, &t\in [-\tau , a\tau ], \\ \varrho _{(a+1)\tau } ^{*}(t), & t\in [a\tau , (a+1)\tau ]. \end{cases}\displaystyle \end{aligned}$$

Then $\mho (a+1)\tau (t, k_{1})$ is the unique solution of Eqs. (16)–(17) on $[-\tau , (a+1)\tau ]$.

Finally, we adopt the operator $\textit{A}_{k_{1}}:C([a_{0}\tau , T];\mathbb{R}^{n})\rightarrow C([a_{0} \tau , T];\mathbb{R}^{n})$ on the range $[a_{0}\tau , T]$ by

$$\begin{aligned} (\textit{A}_{k_{1}}) (t) :=& \mathcal{Y}(0)+ \frac{1}{\Gamma (\lambda )} \int _{0}^{a_{0}\tau }{(t- \zeta )}^{\lambda -1} \mathcal{B}\bigl(\zeta , \mho _{a_{0}\tau }(\zeta , k_{1}), \mho _{a_{0}\tau }(\zeta - \tau , k_{1})\bigr)\,d \zeta \\ &{}+ \frac{1}{\Gamma (\lambda )} \int _{a_{0}\tau }^{t}{(t- \zeta )}^{ \lambda -1} \mathcal{B}\bigl(\zeta , \varpi (\zeta ), \mho _{a_{0}\tau }( \zeta - \tau , k_{1})\bigr)\,d\zeta \quad \forall t\in [a_{0}\tau , T]. \end{aligned}$$

Let $\beta _{a_{0}}$ be a positive constant such that $\beta _{a_{0}}> 2\max_{t\in [a_{0}\tau ,T]}L(t,\mho _{a_{0}\tau }(t- \tau , k_{1}))$. On the space $C([a_{0}\tau , T]; \mathbb{R}^{n})$, we establish the new metric

$$ d_{\beta _{a_{0}}}(\varpi , \varpi _{1}):= \sup _{t\in [a_{0}\tau , T]} \frac{ \Vert \varpi (t)- \varpi _{1}(t) \Vert }{E_{\lambda }(\beta _{a_{0}}t^{\lambda })},$$

and as above, we can prove that the operator $\textit{A}_{k_{1}}$ has a unique fixed-point $\varpi ^{*}$ on $[a_{0}\tau , T]$. Define the function

24 $$\begin{aligned} \mho _{T}(t,k_{1}) := \textstyle\begin{cases} \mho _{a_{0}\tau }(t,k_{1}), & t\in [-\tau , a_{0}\tau ], \\ \varpi ^{*}(t), & t\in [a_{0}\tau , T]. \end{cases}\displaystyle \end{aligned}$$

It is obvious that $\mho _{T}$ is the unique solution of the given IVP (16)–(17) on the time interval $[-\tau , T]$. □

### Derivation of the solution

Here we establish the solution of the given fractional time-delay model (5) by using the well-known Adams–Bashforth–Moulton P-C scheme specified in [46]. A modified version of this method is given in [47]. We know that the numerical algorithm for nondelay and delay systems have their own different dynamics. Also, the stability of nondelay methods can be easily derived but not in delay problems. The advantage of using delay algorithms is that such methods can be also used for solving nondelay problems by setting the time delay parameter equal to zero.

Now consider the following common delay problem for system (5):

25a $$\begin{aligned} &{}^{C}D_{t}^{\lambda } \mathcal{Y}(t)= \mathcal{B}\bigl(t, \mathcal{Y}(t), \mathcal{Y}(t- \tau )\bigr), \quad t\in [0, T], 0< \lambda \leq 1, \end{aligned}$$

25b $$\begin{aligned} &\mathcal{Y}(t)= k_{1},\quad t\in [-\tau , 0]. \end{aligned}$$

Consider the uniform grid $\{t_{\mu }= \mu h : \mu = -\varrho ,-\varrho + 1,\ldots, -1,0,1,\ldots, \mathbb{N}\}$, where *ϱ* and $\mathbb{N}$ are integers such that $h = T/\mathbb{N}$ and $h = \tau /\varrho $. Let

26 $$ \mathcal{Y}(t_{j})= k_{1},\quad j= -\varrho , -\varrho +1, \ldots,-1,0, $$

and consider

27 $$ \mathcal{Y}(t_{j}- \tau )= \mathcal{Y}(jh- \varrho h)= \mathcal{Y}(t_{j- \varrho }),\quad j= 0,1,\ldots,\mathbb{N}. $$

Assume that we have previously established the approximations $\mathcal{Y}(t_{j})\approx \mathcal{Y}(t_{j})$ ($j = -\varrho , -\varrho + 1,\ldots,-1, 0, 1,\ldots,\mu $), and we want to find $\mathcal{Y}(t_{\mu +1})$ using the Volterra integral equation corresponding to Eqs. (25a) and (25b),

28 $$ \mathcal{Y}(t_{\mu +1})= \mathcal{Y}(0)+ \frac{1}{\Gamma (\lambda )} \int _{0}^{t_{\mu +1}}{(t_{\mu +1}- \zeta )}^{\lambda -1}\mathcal{B}\bigl( \zeta , \mathcal{Y}(\zeta ), \mathcal{Y}( \zeta - \tau )\bigr)\,d\zeta . $$

We use approximations $\mathcal{Y}(t_{\mu })$ for $\mathcal{Y}(t_{\mu })$ in (28). The integral in Eq. (28) is derived by using product the trapezoidal quadrature rule. So the corrector equations are

29 $$ \begin{aligned}[b] \mathcal{Y}(t_{\mu + 1})= {}& \mathcal{Y}(0)+ \frac{h^{\lambda }}{\Gamma (\lambda +2)}\mathcal{B}\bigl(t_{\mu +1}, \mathcal{Y}(t_{\mu +1}), \mathcal{Y}(t_{\mu +1}- \tau )\bigr) \\ &{} + \frac{h^{\lambda }}{\Gamma (\lambda +2)}\sum_{j=0}^{\mu }a_{j,\mu +1} \mathcal{B}\bigl(t_{j}, \mathcal{Y}(t_{j}), \mathcal{Y}(t_{j}- \tau )\bigr) \\ ={} & \mathcal{Y}(0)+ \frac{h^{\lambda }}{\Gamma (\lambda +2)}\mathcal{B}\bigl(t_{ \mu +1}, \mathcal{Y}(t_{\mu +1}), \mathcal{Y}(t_{\mu +1-\varrho })\bigr) \\ &{} + \frac{h^{\lambda }}{\Gamma (\lambda +2)}\sum_{j=0}^{\mu }a_{j,\mu +1} \mathcal{B}\bigl(t_{j}, \mathcal{Y}(t_{j}), \mathcal{Y}(t_{j-\varrho })\bigr), \end{aligned} $$

where

$$\begin{aligned} a_{j,\mu +1}= \textstyle\begin{cases} \mu ^{\lambda +1}- (\mu - \lambda ){(\mu + 1)}^{\lambda },&j= 0, \\ (\mu - j+ 2)^{\lambda +1}- 2(\mu - j+ 1)^{\lambda +1}+ (\mu - j)^{ \lambda +1},& 1\leq j\leq \mu , \\ 1,& j= \mu + 1. \end{cases}\displaystyle \end{aligned}$$

The unknown term $\mathcal{Y}(t_{\mu +1})$ appears on every side of (29), and because of nonlinearity of $\mathcal{A}_{1}$, equation (29) cannot be simulated clearly for $\mathcal{Y}(t_{\mu +1})$. So we shift the term $\mathcal{Y}(t_{\mu +1})$ on the right-hand direction by an approximation $\mathcal{Y}^{P}(t_{\mu +1})$, called a predictor. We apply the product rectangle rule in (29) to find the predictor term

30 $$ \begin{aligned}[b] \mathcal{Y}^{P}(t_{\mu + 1})& = \mathcal{Y}(0) + \frac{1}{\Gamma (\lambda )}\sum_{j=0}^{\mu }b_{j,\mu +1} \mathcal{B}\bigl(t_{j}, \mathcal{Y}(t_{j}), \mathcal{Y}(t_{j}- \tau )\bigr) \\ & = \mathcal{Y}(0)+ \frac{1}{\Gamma (\lambda )}\sum_{j=0}^{\mu }b_{j, \mu +1} \mathcal{B}\bigl(t_{j}, \mathcal{Y}(t_{j}), \mathcal{Y}(t_{j- \varrho })\bigr), \end{aligned} $$

where

$$ b_{j,\mu +1}= \frac{h^{\lambda }}{\lambda }\bigl((\mu +1-j)^{\lambda }- (\mu - j)^{\lambda }\bigr). $$

Finally, by all given estimations the corrector terms for the proposed model of equations (6) are

31 $$\begin{aligned}& \begin{aligned} x(t_{\mu + 1}) ={}& x(0)+ \frac{h^{\lambda }}{\Gamma (\lambda +2)}\mathcal{Z}_{1}\bigl(t_{\mu +1}, x(t_{ \mu +1}), x(t_{\mu +1-\varrho })\bigr) \\ &{}+ \frac{h^{\lambda }}{\Gamma (\lambda +2)}\sum _{j=0}^{\mu }a_{j,\mu +1} \mathcal{Z}_{1}\bigl(t_{j}, x(t_{j}), x(t_{j-\varrho })\bigr), \end{aligned} \\& \begin{aligned} y(t_{\mu + 1}) ={} & y(0)+ \frac{h^{\lambda }}{\Gamma (\lambda +2)} \mathcal{Z}_{2} \bigl(t_{\mu +1}, y(t_{\mu +1}), y(t_{\mu +1-\varrho })\bigr) \\ &{}+ \frac{h^{\lambda }}{\Gamma (\lambda +2)}\sum_{j=0}^{\mu }a_{j,\mu +1} \mathcal{Z}_{2}\bigl(t_{j}, y(t_{j}), y(t_{j-\varrho })\bigr), \end{aligned} \\& \begin{aligned} v(t_{\mu + 1}) ={}& v(0)+ \frac{h^{\lambda }}{\Gamma (\lambda +2)} \mathcal{Z}_{3} \bigl(t_{\mu +1}, v(t_{\mu +1}), v(t_{\mu +1-\varrho })\bigr) \\ &{}+ \frac{h^{\lambda }}{\Gamma (\lambda +2)}\sum_{j=0}^{\mu }a_{j,\mu +1} \mathcal{Z}_{3}\bigl(t_{j}, v(t_{j}), v(t_{j-\varrho })\bigr) , \end{aligned} \end{aligned}$$

where

$$\begin{aligned} a_{j,\mu +1}= \textstyle\begin{cases} \mu ^{\lambda +1}- (\mu - \lambda ){(\mu + 1)}^{\lambda },&j= 0, \\ (\mu - j+ 2)^{\lambda +1}- 2(\mu - j+ 1)^{\lambda +1}+ (\mu - j)^{ \lambda +1},& 1\leq j\leq \mu , \\ 1,&j= \mu + 1. \end{cases}\displaystyle \end{aligned}$$

Similarly, the predictor terms are

32 $$ \begin{aligned} & x^{P}(t_{\mu + 1}) = x(0)+ \frac{1}{\Gamma (\lambda )}\sum_{j=0}^{\mu }b_{j,\mu +1} \mathcal{Z}_{1}\bigl(t_{j}, x(t_{j}), x(t_{j-\varrho })\bigr), \\ & y^{P}(t_{\mu + 1}) = y(0)+ \frac{1}{\Gamma (\lambda )}\sum _{j=0}^{ \mu }b_{j,\mu +1} \mathcal{Z}_{2}\bigl(t_{j}, y(t_{j}), y(t_{j-\varrho })\bigr), \\ & v^{P}(t_{\mu + 1}) = v(0)+ \frac{1}{\Gamma (\lambda )}\sum _{j=0}^{ \mu }b_{j,\mu +1} \mathcal{Z}_{3}\bigl(t_{j}, v(t_{j}), v(t_{j-\varrho })\bigr), \end{aligned} $$

where

$$ b_{j,\mu +1}= \frac{h^{\lambda }}{\lambda }\bigl((\mu +1-j)^{\lambda }- (\mu - j)^{\lambda }\bigr). $$

#### Theorem 4

(Error analysis)

*Let us assume that the solution* $\mathcal{Y}(t)$ *of the IVP* (25a)*–*(25b) *satisfies the following constraint*:

33 $$ \Biggl\vert \int _{0}^{t_{\mu +1}}{(t_{\mu +1}- \zeta )}^{\lambda -1} {^{C}}D_{t}^{ \lambda } \mathcal{Y}(t)\,dt- \frac{h^{\lambda }}{\lambda (\lambda +1)} \sum_{j=0}^{\mu }a_{j,\mu +1} {^{C}}D_{t}^{\lambda } \mathcal{Y}(t_{j}) \Biggr\vert \leq Ct_{\mu +1}^{\gamma }h^{\delta } $$

*for some* $\delta >0$, $\gamma \geq 0$, *and suppose that* $\mathcal{B}$ *satisfies the Lipschitz condition for both delay and nondelay variables*, *i*.*e*.,

34 $$ \begin{aligned} & \bigl\Vert \mathcal{B}(t, \mathcal{Y}_{1}, \mathcal{Y}_{d})- \mathcal{B}(t, \mathcal{Y}_{2}, \mathcal{Y}_{d}) \bigr\Vert \leq L_{1}(t, \mathcal{Y}_{d}) \Vert \mathcal{Y}_{1}- \mathcal{Y}_{2} \Vert , \\ & \bigl\Vert \mathcal{B}(t, \mathcal{Y}, \mathcal{Y}_{d1})- \mathcal{B}(t, \mathcal{Y}, \mathcal{Y}_{d2}) \bigr\Vert \leq L_{2}(t, \mathcal{Y}) \Vert \mathcal{Y}_{d1}- \mathcal{Y}_{d2} \Vert . \end{aligned} $$

*Then for* $T > 0$, *we have*

$$ \max_{0\leq j \leq \mathbb{N}} \bigl\vert \mathcal{Y}(t_{j})- \mathcal{Y}_{j} \bigr\vert \leq kh^{\delta },$$

*where* $\mathbb{N} = \frac{T}{h}$, *k* *is a positive constant*, $\mathcal{Y}(t_{j})$ *is the exact solution*, *and* $\mathcal{Y}_{j}$ *is the approximate solution of the IVP* (25a)*–*(25b).

#### Proof

Using the method of mathematical induction, let us assume that the statement is true for $j = 0, 1, 2, \ldots , \mu $. We have

35 $$\begin{aligned} & \bigl\vert \mathcal{B} \bigl(t_{j}, \mathcal{Y}(t_{j}), \mathcal{Y}(t_{j}- \tau )\bigr)- \mathcal{B}(t_{j}, \mathcal{Y}_{j}, \mathcal{Y}_{dj}) \bigr\vert \\ &\quad = \bigl\vert \mathcal{B} \bigl(t_{j}, \mathcal{Y}(t_{j}), \mathcal{Y}(t_{j}- \tau )\bigr)+ \mathcal{B}\bigl(t_{j}, \mathcal{Y}_{j}, \mathcal{Y}(t_{j} - \tau )\bigr) \\ &\qquad {}-\mathcal{B}\bigl(t_{j}, \mathcal{Y}_{j}, \mathcal{Y}(t_{j}- \tau )\bigr)- \mathcal{B}(t_{j}, \mathcal{Y}_{j}, \mathcal{Y}_{dj}) \bigr\vert \\ &\quad \leq \bigl\vert \mathcal{B}\bigl(t_{j}, \mathcal{Y}(t_{j}), \mathcal{Y}(t_{j}- \tau )\bigr)- \mathcal{B}\bigl(t_{j}, \mathcal{Y}_{j}, \mathcal{Y}(t_{j} - \tau )\bigr) \bigr\vert \\ &\qquad {}+ \bigl\vert \mathcal{B}\bigl(t_{j}, \mathcal{Y}_{j}, \mathcal{Y}(t_{j}- \tau )\bigr)- \mathcal{B}(t_{j}, \mathcal{Y}_{j}, \mathcal{Y}_{dj}) \bigr\vert \\ &\quad \leq L_{1} \bigl\vert \mathcal{Y}(t_{j})- \mathcal{Y}_{j} \bigr\vert + L_{2} \bigl\vert \mathcal{Y}(t_{j}- \tau )- \mathcal{Y}_{dj} \bigr\vert \leq (L_{1}+ L_{2})h^{\delta }. \end{aligned}$$

By induction hypothesis Eq. (33) holds for $j = 1, 2, \ldots , \mu $, and now a need to prove the same for $j = \mu + 1$. At the $(\mu +1)$th step,

36 $$ \begin{aligned}[b] & \bigl\vert \mathcal{Y}(t_{\mu + 1})- \mathcal{Y}^{P}_{\mu + 1} \bigr\vert \\ &\quad = \frac{1}{\Gamma (\lambda )} \Biggl\vert \int _{0}^{t_{\mu +1}}{(t_{\mu +1}- \zeta )}^{\lambda -1}\mathcal{B}\bigl(\zeta , \mathcal{Y}(\zeta ), \mathcal{Y}(\zeta - \tau )\bigr)\,d\zeta \\ &\qquad {}-\frac{h^{\lambda }}{\lambda (\lambda +1)}\sum_{j=0}^{\mu }a_{j,\mu +1} \mathcal{B}\bigl(t_{j}, \mathcal{Y}(t_{j}), \mathcal{Y} {dj}\bigr) \Biggr\vert \\ &\quad \leq \frac{1}{\Gamma (\lambda )} \Biggl\vert \int _{0}^{t_{\mu +1}}{(t_{ \mu +1}- \zeta )}^{\lambda -1} {^{C}}D_{t}^{\lambda } \mathcal{Y}(t)\,dt- \frac{h^{\lambda }}{\lambda (\lambda +1)}\sum_{j=0}^{\mu }a_{j,\mu +1} {^{C}}D_{t}^{ \lambda } \mathcal{Y}(t_{j}) \Biggr\vert \\ &\qquad {}+\frac{1}{\Gamma (\lambda )}\frac{h^{\lambda }}{\lambda (\lambda +1)} \sum_{j=0}^{\mu }a_{j,\mu +1} \bigl\vert \mathcal{B}\bigl(t_{j}, \mathcal{Y}(t_{j}), \mathcal{Y}(t_{j}- \tau )\bigr)-\mathcal{B}(t_{j}, \mathcal{Y}_{j}, \mathcal{Y}_{dj}) \bigr\vert \\ &\quad \leq \frac{Ct_{\mu +1}^{\gamma }h^{\delta }}{\Gamma (\lambda )}+ \frac{(L_{1}+L_{2})h^{\lambda +\delta }T^{\lambda }}{\lambda \Gamma (\lambda +2)}, \end{aligned} $$

since

37 $$ \begin{aligned}[b] \sum_{j=0}^{\mu }a_{j,\mu +1} \leq {}&\sum_{j=0}^{\mu }\bigl[ ( \mu - j+ 2)^{\lambda +1}- 2(\mu - j+ 1)^{\lambda +1}+ (\mu - j)^{ \lambda +1} \bigr] \\ &{}\times \sum_{j=0}^{\mu }\bigl[ (\mu - j+ 2)^{\lambda +1}- (\mu - j+ 1)^{ \lambda +1}- (\mu - j+ 1)^{\lambda +1}+ (\mu - j)^{\lambda +1}\bigr] \\ ={}& \biggl[ \int _{0}^{t_{\mu +1}}{(t_{\mu +2}- t)}^{\lambda }\,dt- \int _{0}^{t_{ \mu +1}}{(t_{\mu +1}- t)}^{\lambda }\,dt \biggr] \\ ={}&\frac{1}{\lambda } \int _{0}^{t_{\mu +1}}\bigl[{(t_{\mu +1}- t)}^{\lambda }\bigr]^{\prime }(t)\,dt= \frac{1}{\lambda }t^{\lambda }_{\mu +1} \leq \frac{T^{\lambda }}{\lambda }. \end{aligned} $$

Thus

38 $$ \bigl\vert \mathcal{Y}(t_{\mu + 1})- \mathcal{Y}^{P}_{\mu + 1} \bigr\vert \leq \frac{Ct_{\mu +1}^{\gamma }h^{\delta }}{\Gamma (\lambda )}+ \frac{(L_{1}+L_{2})h^{\lambda +\delta }T^{\lambda }}{\lambda \Gamma (\lambda +2)}. $$

Using Eqs. (35) and (38), we get a bound for the difference between actual and approximate solutions:

39 $$\begin{aligned} & \biggl\vert \mathcal{Y}(t_{\mu + 1})- \biggl[ \mathcal{Y}^{P}_{ \mu +1}+ \frac{h^{\lambda }}{\Gamma (\lambda +2)} \mathcal{B}\bigl(t_{\mu +1}, \mathcal{Y}^{P}_{\mu +1}, \mathcal{Y}_{d(\mu +1)}\bigr) \biggr] \biggr\vert \\ &\quad = \biggl\vert \mathcal{Y}(0)+ \frac{1}{\Gamma (\lambda )} \int _{0}^{t_{ \mu +1}}{(t_{\mu +1}- \zeta )}^{\lambda -1}\mathcal{B}\bigl(\zeta , \mathcal{Y}(\zeta ), \mathcal{Y}(\zeta - \tau )\bigr)\,d\zeta \\ &\qquad {}- \biggl[ \mathcal{Y}^{P}_{\mu +1}+ \frac{h^{\lambda }}{\Gamma (\lambda +2)} \mathcal{B}\bigl(t_{\mu +1}, \mathcal{Y}^{P}_{\mu +1}, \mathcal{Y}_{d(\mu +1)}\bigr) \biggr] \biggr\vert \\ &\quad \leq \frac{1}{\Gamma (\lambda )} \Biggl\{ \Biggl\vert \int _{0}^{t_{\mu +1}}{(t_{ \mu +1}- \zeta )}^{\lambda -1} \mathcal{B}\bigl(\zeta , \mathcal{Y}(\zeta ), \mathcal{Y}( \zeta -\tau )\bigr) \,d\zeta \\ &\qquad {}- \frac{h^{\lambda }}{\lambda (\lambda +1)}\sum _{j=0}^{\mu +1}a_{j,\mu +1} \mathcal{B}\bigl( \zeta _{j}, \mathcal{Y}(\zeta _{j}), \mathcal{Y}(\zeta _{j}- \tau )\bigr) \Biggr\vert \\ &\qquad {}+\frac{h^{\lambda }}{\lambda (\lambda +1)}\sum_{j=0}^{\mu }a_{j,\mu +1} \bigl\vert \mathcal{B}(t_{j}, \mathcal{Y}_{j}, \mathcal{Y}_{dj})-\mathcal{B}\bigl(t_{j}, \mathcal{Y}(t_{j}), \mathcal{Y}(t_{j}-\tau )\bigr) \bigr\vert \\ &\qquad {}+\frac{h^{\lambda }}{\lambda (\lambda +1)} \bigl\vert \mathcal{B}\bigl(t_{\mu +1}, \mathcal{Y}(t_{\mu +1}), \mathcal{Y}(t_{\mu +1}-\tau )\bigr)- \mathcal{B}\bigl(t_{ \mu +1}, \mathcal{Y}^{P}_{\mu +1}, \mathcal{Y}_{d(\mu +1)}\bigr) \bigr\vert \Biggr\} \\ &\quad \leq k h^{\delta }\quad ( \text{from Eqn. (35) and (38) and [48, Theorems 5.1 and 5.2]}), \end{aligned}$$

which gives us the required result

$$ \max_{0\leq j \leq \mathbb{N}} \bigl\vert \mathcal{Y}(t_{j})- \mathcal{Y}_{j} \bigr\vert \leq kh^{\delta }.$$

The proposed error bound specifies the convergence of the method. □

## Graphical simulations

**Figure 1:**
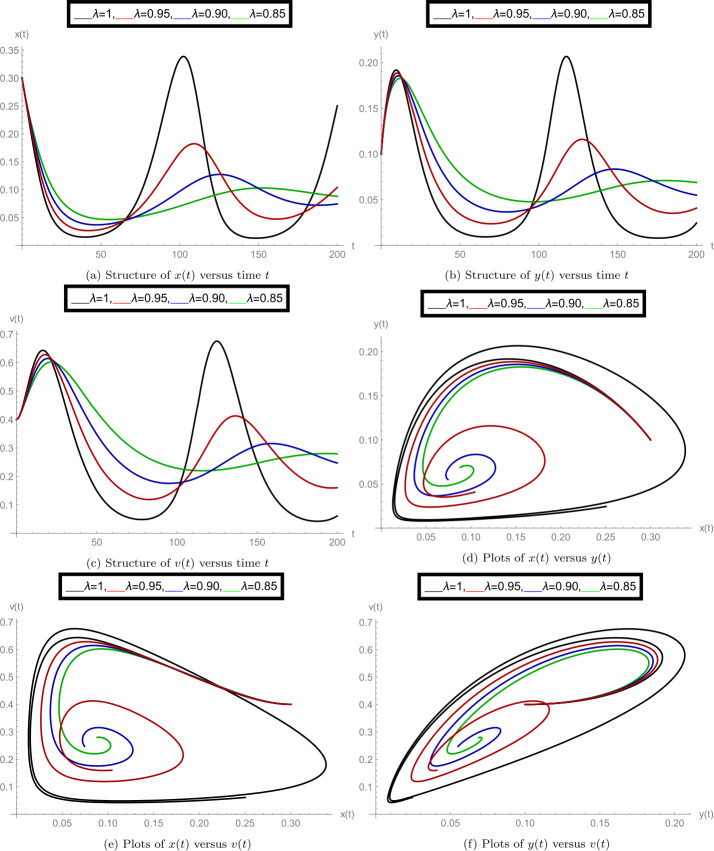
Plots of model classes at distinct fractional-order values *λ* for time delay $\tau =0$

**Figure 2:**
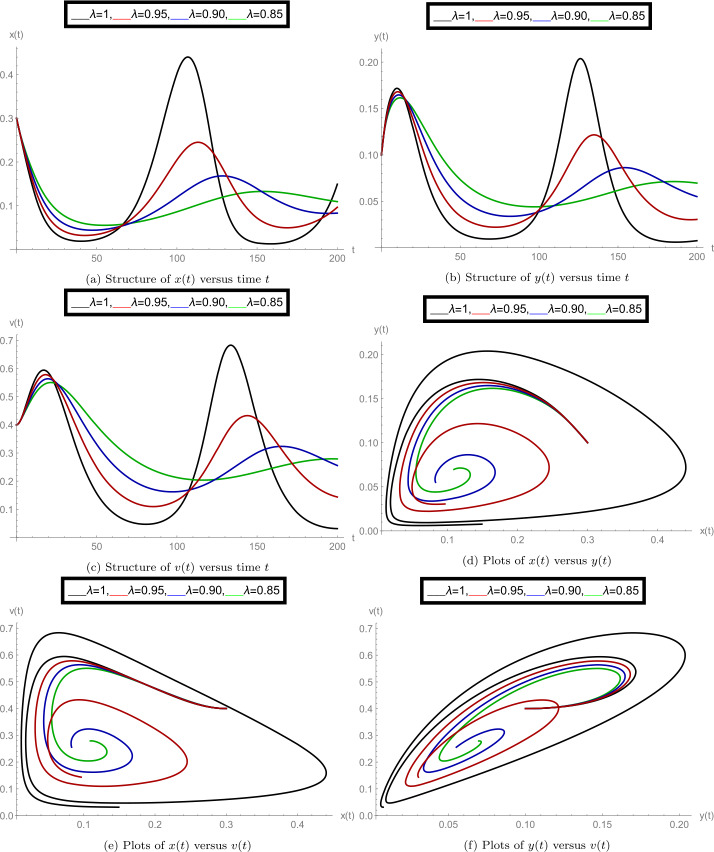
Plots of model classes at distinct fractional order values *λ* for time delay $\tau =2$

**Figure 3:**
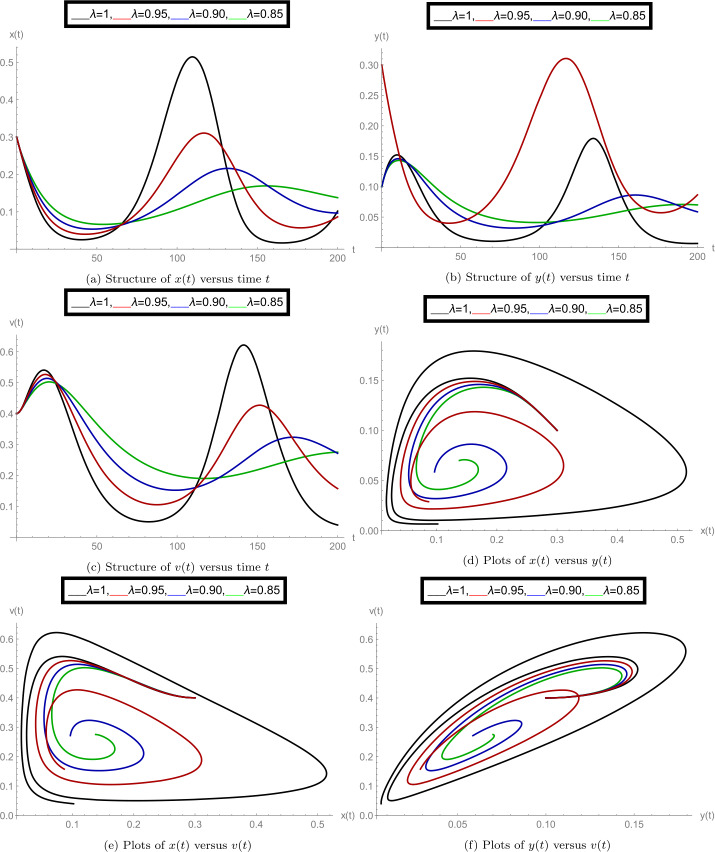
Plots of model classes at distinct fractional-order values *λ* for time delay $\tau =4$

**Figure 4:**
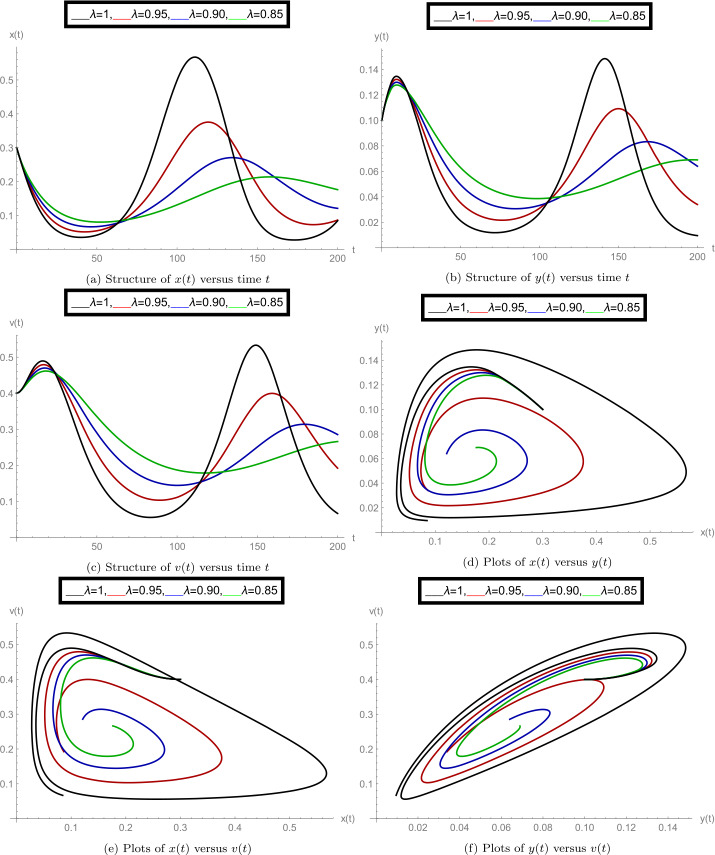
Plots of model classes at distinct fractional-order values *λ* for time delay $\tau =6$

**Figure 5:**
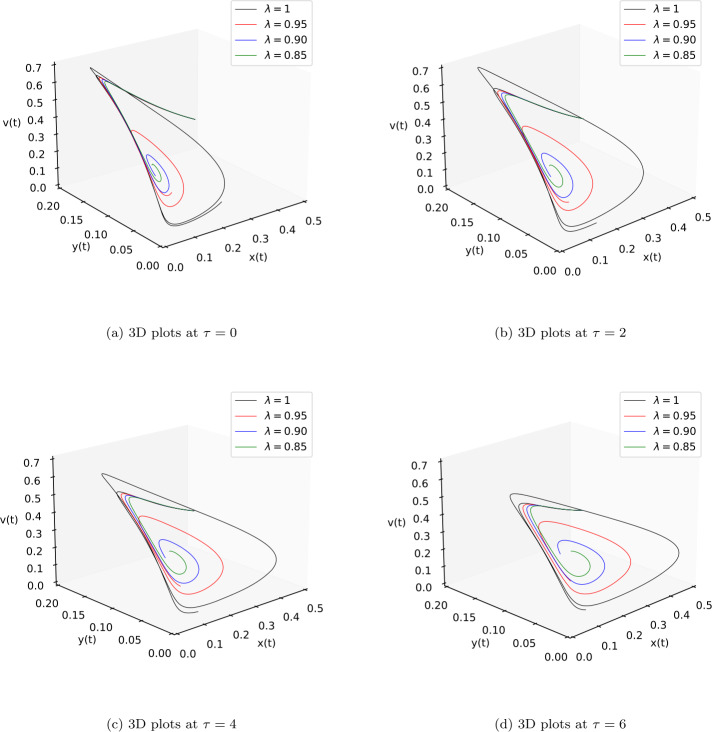
3D Plots of model classes at distinct fractional-order values *λ* and time delays *τ*

**Figure 6:**
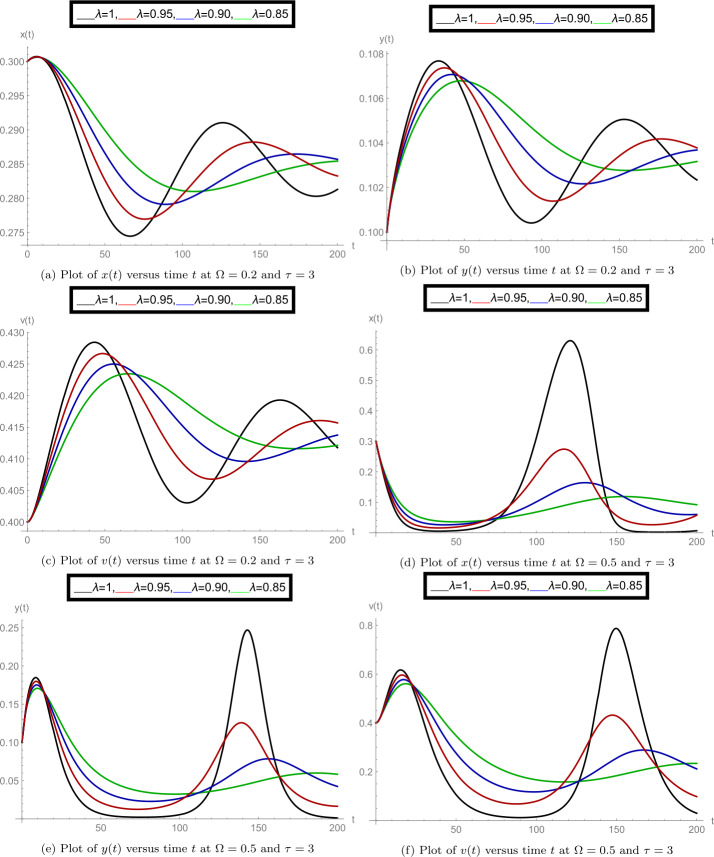
Plots of model classes at distinct fractional-order values *λ* for time delay $\tau =3$ and $\Omega = 0.2, 0.5$

After finishing all theoretical evolutions, we now perform the practical work. In this part of the analysis, we simulate the role of time-delay along with all given parameter values at different fractional orders *λ*. To perform the practical interpretations, we code the above algorithm by using *Mathematica* software. The parameter values we use in these practical simulations are given in Table 1. Figure 1 shows the dynamics of the model classes at different Caputo operator values of orders $\lambda = 1, 0.95, 0.90, 0.85$. In this group, Fig. 1a justifies the behavior of plenty of susceptible plants $x(t)$, Fig. 1b shows the nature of plenty of infected plants $y(t)$, and Fig. 1c shows the dynamics of infected vector $v(t)$ with respect to the time variable *t*. In Figs. 1d, 1e, 1f, compatible dynamics of $x(t)$ versus $y(t)$, $x(t)$ versus $v(t)$, and $y(t)$ versus $v(t)$ are exemplified, respectively. We observed that for the given time rage, the oscillations occur in the population of susceptible, infected plants, and in the infected vectors, but when the derivative order reduces, then the oscillations also decrease, which justifies the boundedness of fractional-order solutions. Also, the 2D figures represent the compatible structures of the model classes, which clarify that when the infected vectors reduce, then the infectious plants definitely decrease. In this case, we fixed the time-delay parameter $\tau =0$, which means that there is no delay in the model. Here we come to considering some delay in the model. First, we take the time-delay $\tau = 2$ and exemplify the group of Fig. 2. In this group, Fig. 2a defines the behavior of plenty of susceptible plants $x(t)$, Fig. 2b shows the nature of plenty of infected plants $y(t)$, and Fig. 2c shows the dynamics of infected vector $v(t)$ with respect to the time variable *t*. In Figs. 2d, 2e, and 2f, the compatible dynamics of $x(t)$ versus $y(t)$, $x(t)$ versus $v(t)$, and $y(t)$ versus $v(t)$ are exemplified, respectively. Here we observed that the dynamics of the oscillations is probably same for $\tau =0$ and $\tau =2$, but the infectious plant population is slightly higher at $\tau =2$. For observing the role of time-delay more clearly, we plotted the figures at two more different values of *τ*. In Figs. 3 and 4, we gave the graphical interpretations of model classes at $\tau =4$ and $\tau =6$, respectively. Here the dynamical changes in the model can be easily observed at various fractional-order values. In Fig. 5, we plotted the 3-D graphics of given classes compatible to each other at different fractional-order values. In Figs. 5a, 5b, 5c, and 5d, 3-D trajectories at time delays $\tau = 0$, $\tau = 2$, $\tau = 4$, and $\tau = 6$ are exemplified, respectively, for different fractional-order Caputo derivatives. From these 3-D plots we see that when the time-delay increases, the flatness in the phases increases. In all above simulations, the infection rate of plant Ω was fixed at $\Omega = 0.4$. Here we simulate some graphs at different values of infection rate to explore the role of parameter Ω on the classes of the model. In this matter, Fig. 6 is devoted to the structure of the proposed model at $\Omega = 0.2, 0.5$. In Figs. 6a, 6b, and 6c, the behavior of $x(t)$, $y(t)$, and $v(t)$ at infection rate $\Omega = 0.2$ is exemplified, respectively. In Figs. 6d, 6e, and 6f, the nature of $x(t)$, $y(t)$, and $v(t)$ at infection rate $\Omega = 0.5$ is demonstrated, respectively. In these simulations, the time-delay parameter is fixed at $\tau =3$. These simulations show that oscillations occur in each case and at every value of fractional order. As compared to the basic classical model exemplified in [40], the given fractional model is more effective and reliable in the graphical interpretation point of view. Also, biologically, the fractional-order model showed a very deep dynamics of the mentioned disease in plants (the way of spreading, population of infectious plants, and amount of infected vectors). Here the trajectories give us enough extension to simulate the model structure at various cases. Also, the given algorithm is reliable to expand for the large time interval $[0, T]$. The idea of using equal dimensions on both sides of the proposed fractional model (5) makes this study more accurate as compared to the studies where researchers do not follow the same dimensionality when generalizing the integer-order models to fractional sense.

## Conclusions

In the given research analysis, we have simulated a mathematical structural model involving the infection rate of Beddington–DeAngelis functional response type and analyzed the structure of vector-borne plant epidemic. The solution existence techniques for time-delay fractional models are totally different from the general nonclassical models. So we have proved that for the given delay mathematical model, a unique global solution exists with some specific restrictions in which the Lipschitz condition is only necessary for the nondelay variable. The Adams–Bashforth–Moulton P-C algorithm has been used to find the solution of the given plant disease model. We have given a brief graphical interpretation of the proposed solution. A number of novel results are demonstrated from the given graphical observations. In 3-D plots, we observed how the flatness in the graphics does change when the fractional order varies. We have also checked the role of time delay on the proposed plant disease dynamics and the effects of infection rate on the population of susceptible and infectious classes. In future, this paper can be further expanded to derive the optimal control strategies for such vector-borne plant disease. Also, any specific plant disease can be studied by different data fittings. Some other fractional derivatives can be used to simulate the given model dynamics.

## Data Availability

The data used in this study are mentioned/available in the manuscript.
